# A Treatise on Human Physiology

**Published:** 1861

**Authors:** 


### BOOK NOTICE.

A TREATISE ON HUMAN PHYSIOLOGY, BY JOHN C. DALTON, Jr., M. D., PROFESSOR OF PHYSIOLOGY AND MICROSCOPIC ANATOMY IN THE COLLEGE OF PHYSICIANS AND SURGEONS, NEW YORK.

We take pleasure in adding our mite of praise to this invaluable and universally popular work. As a text book—in our estimation, it is superior to any in use. While the language is elegant and classical, the author's meaning is everywhere apparent on the surface.

What adds very greatly to the value of the work, is the author's account of his repetition of experiments—both new and old—with a description of the modus operandi of their performance, thereby giving to his labors the freshness of originality—a merit sadly wanting in most works on this subject.

We observe that our author fully sustains M. Claude Bernard's theory of the glyco-genic function of the liver—a speculation which has been roughly handled of late by F. W. Pavy, M. D., Prof. of Phys. in Guy's Hospital, Lecturer, etc., London.

With regard to the sugar-making function of the Liver Prof. Dalton states that

"It has long been known that sugar may be abundantly secreted under some circumstances when no vegetable matters have been taken into the food. The milk, for example, of all animals, carnivorous as well as herbivorous, contains a notable proportion of sugar; and the quantity thus secreted during lactation is in some instances very great. In the human subject, also, when suffering from diabetes, the amount of saccharine matter discharged with the urine has often appeared to be altogether out of proportion to that which could be accounted for by the vegetable substances taken as food.

The experiments of Bernard, the most important of which we have repeatedly confirmed in common with other investigations, show that in these instances most of the sugar has an internal origin, and that it first makes its appearance in the tissue of the liver."

If a carnivorous animal, as, for example a dog or cat, be fed for several days exclusively upon meat, and then killed, the liver alone of all the internal organs is found to contain sugar among its other ingredients.

Again he observes that

"The sugar which is found in the liver after death, is a normal ingredient of the hepatic tissue. It is not formed in other parts of the body, nor absorbed from the intestinal canal, but takes its origin in the liver itself; it is produced, as a new formation, by a secreting process in the tissue of the organ."

Now while we are ready to affirm from experiment and observation that sugar is present in the liver after death, yet we take the liberty of introducing some of the experiments of Dr. Pavy, showing we think, that there is at least room for doubt as to its presence in any appreciable quantity during life.

Dr. Pavy states that

"Under the mode of procedure that used to be adopted, it certainly seemed satisfactorily proved, that whilst the blood in the arterial system was almost free from sugar, that in the right cavities of the heart—that, in part, between the liver and the lungs—was highly charged with it. In obtaining these results, a specimen of blood was collected from an artery and examined. The life of the animal was then destroyed, and by an incision into the right auricle or ventricle their contents were procured. Now unless certain precautions are observed, the necesssity for which was not formerly known, such a method of experimenting must inevitably lead to a fallacious physiological conclusion. The knowledge required is of the state belonging to life, and this we fail to obtain unless we operate on blood, cardiac as well as arterial, that is collected at the very instant of death.

Again he remarks that

"I was first led to observe this variation in the condition of the blood that take place immediately after death, whilst experimenting on the injection of blood through some artificially inflated lungs, for the purpose of imitating what occurred during life. I had been in the habit at first of employing blood collected from the right side of the heart after death, but afterwards procured it from the living ventricle, by a process, so to speak of cardiac catheterism, an operation that is easily performed without leading to any sensible injury to the animal. An instrument is used that is specially curved for the purpose. It is introduced into the right jugular vein and passed down through the superior cava into the heart. I was astonished to find the blood thus collected present a totally different behaviour to what had hitherto been considered as belonging to it, from collecting and examining it after death. I had been accustomed to meet with a strong reaction of sugar as belonging to right ventricular blood, but in that withdrawn during life, I failed to discover more than a scarcely appreciable indication of the presence of the sacharine principle."

Again Dr. Pavy says that

"When blood is collected from the right side of the heart as in an ordinarily conducted examination after death, it yields a strong indication of the presence of sugar. In fine quantative analysis I found the proportion of sugar to vary from half a grain to one grain per cent. in defibrinated blood. When collected on the other hand, duriug life, under natural circumstances, the amount of sugar is certainly not more than is entertained in the arterial system.

Dr. Pavy disposes of the presence of sugar in the liver after death in this manner:

"It now occurred to me to examine closely the chemical relations of Hepatine as far as regarded its transformation into sugar. But, in the first place, knowing that hepatine was convertible into sugar by a kind of fermentation, a foreign substance—a strong solution of sulphate of soda, was injected into the liver immediately after death, to see if it influenced this fermentation and altered the amount of sacharine matter found. The result obtained induced me to proceed. Upon the injection of potash being performed, it was in vain I sought for the accustomed behavior of the liver. But the fact required collateral proof, as it led to so important a conclusion. That the potash had not destroyed the sugar, but simply prevented its formation, was shown by practising the injection upon a liver that had been allowed to remain a short time after death, to permit the production of sugar to take place. The result, under these circumstances, was, that the presence of sugar was as readily discoverable as if the injection of the alkali had not been employed."

Dr. Pavy found that high and low degrees of temperature arrested the formation of sugar in the recent liver. He says:

"The life of the animal being suddenly destroyed, the abdomen is immediately opened and a piece of liver excised as hastily as possible and plunged into the freezing mixture in which it is aftewards moved about; in a short time the liver is frozen hard. On removal it is cut in three slices, reduced to a pulp in a mortar and thrown, a little at a time, into a small quantity of water contained in a capsule, which is to be kept thoroughly boiling during the process. If the specimen were allowed to pass through a gradual elevation of temperature to obtain the decoction for testing, the experiment would be vitiated, as sugar would be formed during the process of preparation. The liquid procured is a concentrated decoction of the liver. It contains plenty of hepatine, but gives no indication or, at very most, the merest trace of sugar.

So we are led to conclude from the result of Dr. Pavy's experiments that liver sugar is derived from hepatine by a process of fermentation occurring after death, and that this process can be arrested at will by using means that will prevent fermentation.

We find this the second edition is improved and enlarged by the addition of an entire chapter on the special senses, which were only incidentally treated of in the former edition, with many other additions of a valuable and interesting character.

In conclusion, we would most earnestly recommend this volume to the Medical public, as one that should have its place in every well regulated Medical library. F.

